# Editorial: Non-coding RNAs as emerging players in the development, diagnosis, and treatment of cancer

**DOI:** 10.3389/fmolb.2024.1453268

**Published:** 2024-07-23

**Authors:** Upendarrao Golla, Ilyas Chachoua

**Affiliations:** ^1^ Department of Medicine, Division of Hematology and Oncology, Pennsylvania State University College of Medicine, Hershey, PA, United States; ^2^ Department of Molecular Biology and Genetics, Bilkent University, Ankara, Türkiye

**Keywords:** lncRNA, miRNA, circRNA, cancer, diagnosis, metastasis, drug resistance, RNA therapeutics

Non-coding RNAs (ncRNAs), including microRNAs (miRNAs), small interfering RNAs (siRNAs), long non-coding RNAs (lncRNAs), and circular RNAs (circRNAs) represent a significant part of the human transcriptome. These RNAs were disregarded as “junk” due to their inability to code for functional proteins. However, it is evident from the last 2 decades of research that these ncRNAs play a critical role in gene regulation at both transcriptional and post-transcriptional level and control various biological pathways, cellular physiology, developmental processes, and disease pathogenesis, including cancer. Recently, these ncRNAs attracted extensive attention due to their specific expression and promising applications in the diagnosis, prognosis, and treatment of various cancers (Piergentili et al., 2022; Uppaluri et al., 2023). In this Research Topic, we are excited to present four outstanding articles encompassing both original research and reviews discussing recent advances in the field with an emphasis on ncRNA’s role in the development, diagnosis, and treatment of cancer and resistance to anticancer therapies.

Cancer is a global burden, with 10 million deaths in 2020, and lung cancer being the most commonly occurring cancer, accounting for 12.4% of the total new cases and 18.7% of the total cancer deaths. Most lung cancer patients were diagnosed in the middle and late stages of cancer due to a lack of early clinical symptoms. Cao et al. conducted a meta-analysis of the existing literature and presented a systematic review of the diagnostic potential of exosome-carried ncRNAs (miRNAs, lncRNAs, circRNAs) in lung cancer. Exosomes are small cell-derived lipid bilayer vesicles with 30–150 nm in diameter and distributed in various body fluids, including blood, saliva, urine, and breast milk. Exosomes have been shown to mediate intercellular communication and play a crucial role in the progression of cancer and tumor microenvironment (TME) (Jia et al., 2022). Due to their ability to transport various biomolecules (proteins, DNA, mRNAs, ncRNAs), exosomes serve as novel noninvasive biomarkers for early diagnosis of cancer. The authors analyzed 60 miRNAs, 9 lncRNAs, and 14 circRNAs reported in 68 studies. Based on the combined selectivity and specificity, authors concluded that exosomal ncRNAs are effective in the diagnosis of lung cancer and may serve as clinical diagnostic biomarkers in the near future.

In recent years, the aberrant expression of ncRNAs has been linked to the development and progression of multiple cancers and metastasis. Among ncRNAs, antisense lncRNAs that are transcribed from the opposite strand of genes with either protein-coding or non-coding function are reported to play an important role in the process of tumor initiation, development, invasion, and migration (Liu et al., 2021; Golla et al., 2022). Due to nucleotide sequence complementarity, antisense lncRNAs regulate the corresponding sense gene. In this Research Topic, Odaka et al. presented an original research article discussing the role of Ephrin type-A receptor 2 antisense RNA (EPHA2-AS) in the proliferation of breast adenocarcinoma cells through the Ras signaling pathway mediated by MAPK8/JNK1, MAPK9/JNK2-NFATC2/NFAT1 and JUND. In their previous research, authors showed that EPHA2-AS has two splice variants (EPHA2-AS1/2) that were constitutively expressed in various breast cancer cell lines and patient samples and regulate EPHA2 mRNA levels (Okuyama et al., 2020). EPHA2 is a receptor tyrosine kinase (RTK) that has an oncogenic role in multiple cancers, including breast and lung cancer, glioblastoma, and melanoma. Epha2 signaling has a key role in TME, and harnessing antitumor immunity was found to be a promising strategy against Epha2-expressing cells. The authors showed that the triple-negative breast cancer (TNBC) cells treated with AHCC, a proprietary and standardized extract of cultured mycelia of *Lentinula edodes,* exhibited a reduced expression of EPHA2-AS1/2 and EPHA2, leading to slower proliferation and migration. So, the article highlights the functional role of EPHA2-AS in breast adenocarcinoma progression and treatment.

In addition to the promising role in cancer diagnosis, development, and progression, ncRNAs emerged as a potential class of therapeutics for multiple diseases, including cancer. Since the FDA approval of the siRNA drug Patisiran in 2018 for treating hATTR (hereditary transthyretin amyloidosis), several ncRNA therapeutics are undergoing clinical trials for the treatment of various diseases. However, small ncRNA-based therapeutics pose several challenges due to limited stability and on-target efficiency. A mini review from Yang et al. provided an updated list of ongoing clinical studies with small ncRNAs as therapeutics and highlighted recent technologies that offer promising delivery of small ncRNA cancer therapeutics for successful pre-clinical and clinical application. Also, the authors discussed the immunomodulatory effects of small ncRNAs and their potential as cancer immunotherapy.

Recent studies showed that ncRNAs, including lncRNAs, are key regulators of essential biological processes involved in the modulation of drug response and the development of resistance to cancer treatments (Wang et al., 2020; Zhou et al., 2022). In this regard, Cantile et al. provide an up-to-date review on the crucial role of lncRNA homeobox transcript antisense intergenic RNA (HOTAIR) in anticancer therapy (Chemo/radiotherapy) resistance. The authors discussed various mechanisms through which oncogenic lncRNA HOTAIR promotes cell proliferation, invasion, and metastasis in different cancers. Majorly, the authors focused on detailing various mechanisms through which HOTAIR lncRNA modulates anticancer therapies and develops resistance to chemotherapy, radiotherapy, endocrine therapy, and targeted therapies such as anti-HER2, anti-epidermal growth factor receptor (EGFR), anti-human epidermal growth factor receptor 2 (HER2), anti- Anaplastic kinase lymphoma (ALK), and multi-target tyrosine receptor kinase inhibitors (TRKI). While further research is necessary to understand the role of HOTAIR lncRNA in the alteration of immunotherapy response and resistance, the authors provided a comprehensive overview of current research on the immunomodulatory effects of HOTAIR lncRNA in this review and emphasized the clinical importance of it as a predictive biomarker for the treatment of cancer patients.

In summary, the work compiled in this Research Topic highlights the importance of ncRNAs as independent or auxiliary biomarkers in cancer diagnosis and therapeutic targets. Furthermore, small ncRNA-based therapeutics need next-generation formulations for effective *in vivo* delivery. The role of ncRNAs in the modulation of existing and future anticancer agents’ efficacy and acquired drug resistance needs further mechanistic investigations. It aims to encourage researchers to explore the role of ncRNAs in different diseases, including cancers and immunomodulation, with a potential to develop novel effective therapies for the treatment of cancer.
